# Comment on “volume-outcome relationship on survival and cost benefits in severe burn injury: a retrospective analysis of a Japanese nationwide administrative database” (Endo et al., *Journal of Intensive Care* 2019)

**DOI:** 10.1186/s40560-020-00462-3

**Published:** 2020-07-08

**Authors:** Akinori Osuka, Daiki Miyao, Yuichi Kuroki

**Affiliations:** Department of Trauma, Critical Care & Burn Center, Japan Community Healthcare Organization Chukyo Hospital, 1-1-10 Sanjo, Minamiku, Nagoya, Aichi 457-8510 Japan

**Keywords:** Burn, In-hospital mortality, Size of burn center

## Abstract

This is a critical comment on the paper by Endo et al. on the volume-outcome relationship on survival and cost benefits in severe burn injury which addresses biases related to patient transfer and burn severity assessment.

## Comment

We read with interest the manuscript titled “Volume-outcome relationship on survival and cost benefits in severe burn injury: a retrospective analysis of a Japanese nationwide administrative database” published by Endo et al. in the *Journal of Intensive Care* in January 2019 [1]. The authors conclude that high burn patient volume was significantly associated with increased in-hospital mortality. We would like to offer some critical comments on the conclusions made from the results of this study.

First, in the methods section, the authors stated that they excluded patients who were transferred to another hospital within 3 days of admission. Further, Figure S7 shows that compared to Figure 2, in-hospital mortality is markedly increased in low-volume burn centers. Figure S7 shows the association between annual severe burn patient volume and the adjusted risk of in-hospital survival among patients who survived for more than 2 days after admission. If a low-volume burn center transfers severe burn patients who would have died within 2 days to a high-volume center, this could explain the differences between Figures 2 and S7. In other words, low-volume centers care for patients with severe burns within 3 days who were not counted in their death toll because they were transferred to a different hospital, whereas high-volume centers care for patients with severe burns until the end, which increases their in-hospital mortality rate.

Second, assessing the size of a burn has proven difficult. Physicians who are unfamiliar with burn sizing tend to overestimate it and underestimate it less often [2]. This can also contribute to a higher life-saving rate in low-volume centers.

In addition, it has been shown that the time until the death of patients with severe burns who survive the resuscitation period is more than 1 week [3]. Toxic shock syndrome, a known fatal complication, usually develops around 3 to 5 days after injury [4], and burn-induced sepsis usually develops 1 week after injury. Such late occurrence of sepsis is more specific to burn injuries than to other trauma patients [5]. High-volume burn centers often accept these infectious burn patients from low-volume centers after the third day of injury. Such patients are counted as in-hospital survivors in the low-volume centers, which may contribute to their lower in-hospital mortality rates, lower healthcare costs, and higher number of hospital-free days.

In summary, the data and statistical analysis used in this study were adequate, but there were potential biases due to the transfer and selection of severely injured patients. Bias can originate from the “transfer out” of patients and selection bias due to choosing patients with a severe systemic condition who might affect survival within 2 days (these patients would be admitted more often to high-volume burn centers), which was not accounted for by the risk adjustment based on burn severity. This is why the discrepancy between the original analysis in Figure 2 and the sensitivity analysis in Figure S7 appears to be so obvious. Further data on hospital center-level transfer rates could be obtained by supplementing the data with sensitivity analyses of transfer rates. We suggest that an appropriate sensitivity analysis to visualize these potential biases requires the following to be performed: (1) remove both the patients who were transferred in from outside the institution and those who were transferred out (so these patients cannot be counted in the survival figures) and (2) exclude patients who died within 2 days as the quality of burn care would be best reflected by the care administered to those patients who survive more than 2 days. This analysis would account for the initial severity of illness that reflects the imminent risk of death, for example, carbon monoxide or cyanide poisoning or cardiac arrest on the scene.

In conclusion, from the setting of this study, the authors cannot conclude that high burn patient volume was significantly associated with an increase in in-hospital mortality.

## Data Availability

Not applicable.
